# Multiple isolated small bowel perforations following blunt abdominal trauma

**DOI:** 10.1016/j.ijscr.2018.10.023

**Published:** 2018-10-19

**Authors:** João Louro, Miguel Albano, Teresa Caroço, Luís Reis, Carlos C. Almeida

**Affiliations:** a Centro Hospitalar e Universitário de Coimbra, Hospital Geral, Cirurgia C Department, Portugal; b IPOFG — Instituto Português de Oncologia de Coimbra Francisco Gentil, E.P.E, Portugal

**Keywords:** Case report, Abdominal trauma, Small bowel injury, Surgery

## Abstract

**Introduction:**

Small bowel injury is uncommon after blunt abdominal trauma. Repeated clinical assessment is important, especially when investigative imaging is negative.

**Case presentation:**

39-year-old male presented to the emergency department following a blunt abdominal trauma. No initial hemodynamic abnormalities were found. Abdominal CT scan was negative for small bowel perforation. Repeated clinical assessment revealed increasing abdominal pain with tachycardia, and an emergent laparotomy was undertaken. Four grade II and one grade I small bowel perforations were found, all repaired with interrupted sutures. Patient was discharged home on day 7.

**Discussion/Conclusion:**

The diagnosis of small bowel injury is difficult and a low threshold of suspicion is crucial to reduce morbidity and mortality. Hemodynamic instability or abdominal tenderness after blunt abdominal trauma are indications for immediate surgical exploration, despite negative imaging findings. Serial clinical assessment is the main decision tool to perform an abdominal exploration.

## Highlights

• Small bowel injury following blunt abdominal trauma is rare.
• Clinical assessment is crucial to achieve an early diagnose, thus reducing morbidity and mortality.
• Hemodynamic instability and/or abdominal tenderness after blunt abdominal trauma raise the need for emergent laparotomy.

## 1 Introduction

Small bowel injury (SBI) is uncommon after blunt abdominal trauma occurring in 1% of the patients. Only 0,3% of all blunt abdominal traumas will have a small bowel perforation (SBP) [1]. The diagnostic approach to SBI is not yet well described and widely accepted because of its rarity and lack of imaging specific indicators [2].

Early diagnosis is of paramount importance to reduce morbidity and mortality [3]. There is an increasing acceptance of non-operative management in hemodynamically stable patients with blunt abdominal trauma and solid organ injury [4]. A laparoscopic approach in abdominal trauma can decrease the chance of a negative laparotomy and allow a diagnostic and therapeutic option with reduced morbidity [5]. However, in blunt abdominal trauma the formal indications for minimal invasive approach are not yet established, and several factors related to the patient and surgical team must be taken into account in decision-making process [6].

A low threshold of suspicion and serial clinical evaluation (noninvasive hemodynamic monitoring, intermittent abdominal examination, serial arterial blood gas analysis) are of extreme importance in order to anticipate peritoneal spillage of enteric fluid, and reduce morbidity and mortality associated with delayed diagnosis, especially when imaging findings are negative for SBP [3]. Since the rate of false negatives when using only CT scan can be as high as 15%, relying only on imaging findings can delay the diagnosis of SBI [7].

The authors present a case of a 39-year-old male with multiple small bowel perforations following a blunt abdominal trauma. He was submitted to emergent laparotomy based on the clinical evaluation, which was contrasting with a normal abdominal CT scan. The main purpose of this case report is to highlight the importance of a serial clinical assessment.

## 2 Presentation of case

**Fig. 1:**
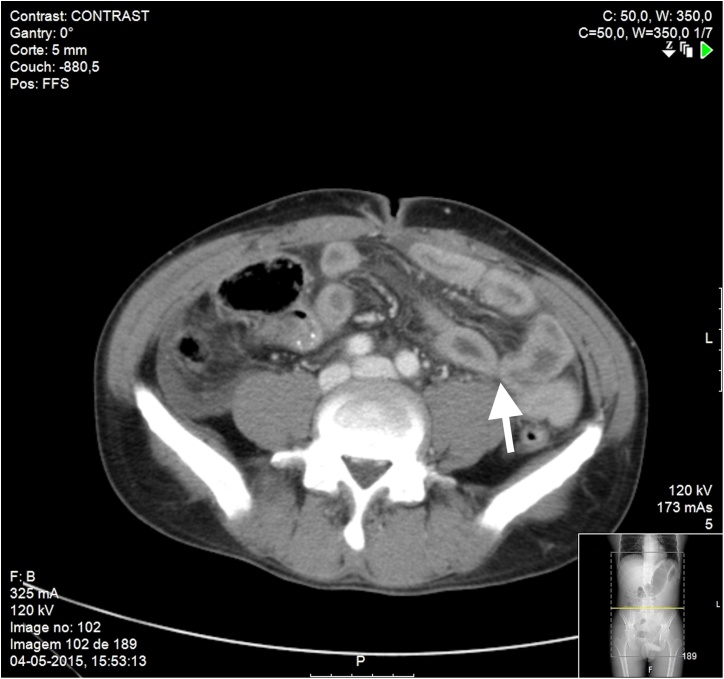
Abdominal CT: small bowel thickening (arrow).

A 39-year-old white male was brought in by ambulance to the Emergency Department of a Tertiary Hospital, 1 h after suffering a blunt abdominal trauma. The patient presented hemodynamic stability without hypotension, tachycardia or tachypnea (blood pressure of 140/99 mmHg, cardiac frequency 75–81 bpm and 12 rcm). The abdomen was plain and skinny without wounds or bruises, with diffuse tenderness without guarding. The abdominal eco-FAST showed a small volume of inter-loop fluid “trapped” in the left flank. Thorax X-ray showed no pneumoperitoneum. Blood work showed no anemia (hemoglobin: 14.7 g/dl, hematocrit: 43,8%). An abdominal CT was performed, which revealed intramural small bowel hemorrhage without pneumoperitoneum (Fig. 1). Analgesic medication was given, and a serial clinical monitoring was conducted. During the first hour there was an increase in abdominal pain resistant to painkillers and despite adequate crystalloid infusion a tachycardia (110 bpm) without hypotension was arisen, along with sweaty and cold extremities. The presence of hemodynamic instability raised the suspicion of abdominal organ injury (Class II of the hemorrhagic shock classification).

**Fig. 2:**
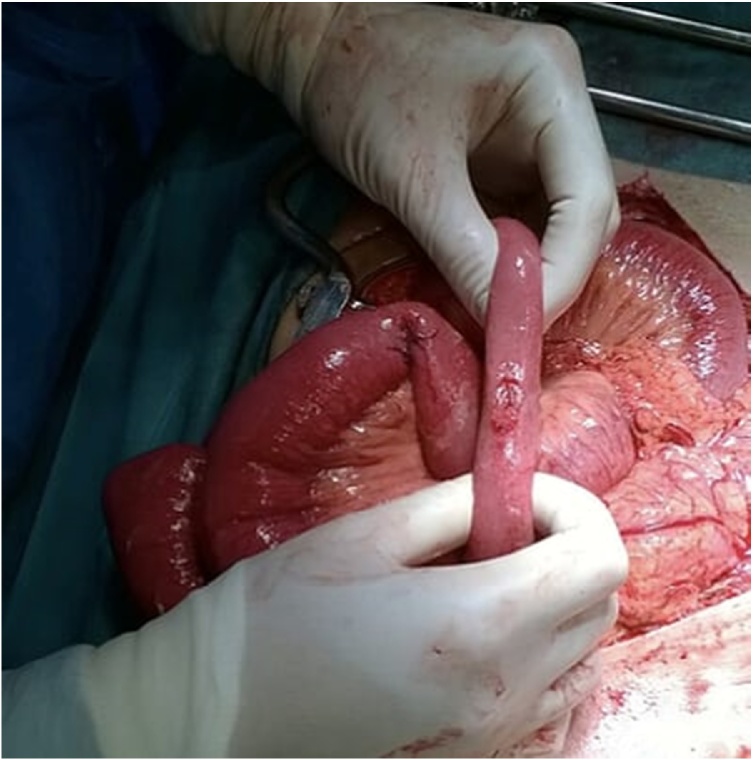
Small bowel perforation, antimesenteric border.

Emergent laparotomy through a midline incision was performed. A small amount of free hematic fluid was found, as well as adhesions. The small bowel was carefully inspected from the ligament of Treitz up to the ileocecal valve. Four grade II jejunoileal perforations and one grade I seromuscular partial thickness laceration without perforation were found. One of the grade II lacerations was located at the mesenteric border of the small bowel (Fig. 2).

The jejunoileal lacerations were repaired with interrupted serosubmucosal Lembert 3/0 absorbable sutures and covered with a sealant matrix coated with humanfibrinogen and thrombin (Tachosil^®^). The peritoneal cavity was washed-out with warm saline solution, and three Jackson-Pratt drains were placed in the peritoneal cavity (one on each left and right parietocolic gutters, and one in the Douglas pouch). Surgery was uneventful and the patient was transferred to the surgical ward.

There was no significant abdominal post-operative pain. The drains were removed on the 3rd and 5th day, and oral diet initiated on the 5th post-op day. The patient was discharged home on day 7. After one-year follow-up the patient is asymptomatic and without complications.

## 3 Discussion

**Fig. 3:**
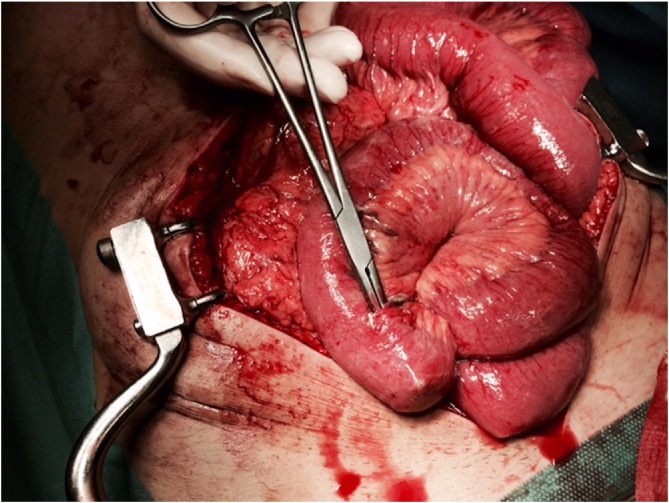
Small bowel perforation, mesenteric border.

Small bowel perforations following blunt abdominal trauma are rare (0,3%), and even rarer when isolated [1]. To the best of the authors’ knowledge, this is the first described case of multiple isolated small bowel perforations following blunt abdominal trauma with a plank (Fig. 3). The time delay between the trauma and surgical management is of paramount importance: if greater than 24 h, mortality increases 4 times compared with less than 8 h. Since the rate of false negatives when using only CT can be as high as 15%, relying only in imaging findings can delay the diagnosis of SBI [7].

Abdominal trauma with SBI can occur due to compression or deceleration forces. A direct blow to the abdomen with external compression of the small bowel against a fixed object increases its intraluminal pressure, causing rupture. On the other hand, deceleration forces cause sheering of the small bowel near its fixing point, such as the ligament of Treitz, ileocecal valve, or the mesentery, which might cause a perforation. In this case a combination of both may have been present since the patient had adhesions from the previous surgery, which can be points of bowel rupture together with compression forces [8].

If a small bowel perforation is confined or temporarily covered, or only liquid is leaking, pneumoperitoneum may be absent. Kahn et al. suggest that, in the presence of intra-abdominal fluid with no solid organ injury, there should be high suspicion of bowel or mesenteric injury. However, the authors state that the presence of free peritoneal fluid is not a specific finding of bowel injury [9]. Moreover, the absence of free gas under the diaphragm does not exclude the presence of small bowel perforation [10]. In the present case, was the serial clinical evaluation that leads the surgical team to perform an emergent laparotomy. This fact highlights the importance of serial clinical surveillance of abdominal trauma patients as a major tool in diagnosis and treatment process, instead of relying only on imaging findings to make those decisions.

The laparoscopic approach should be considered in hemodynamically stable patients who suffered blunt abdominal trauma with suspected intra-abdominal organ injuries, and equivocal imaging findings. Furthermore, it has the advantage of avoiding negative laparotomies, allows diagnostic and therapeutic measures, reduces post-operative morbidity with shorter hospital stay and fastest recovery [5]. However, other factors must be taken into account, such as patient related factors, surgical team laparoscopy experience and occasional inadequate injury visualization and missed injury. A published overview focused on the practical aspects of the potential complications of laparoscopy in abdominal trauma (ref to Kindel et al.) states that when laparoscopy exploration is performed following previous abdominal procedures, there is a 12% rate of failure to achieve pneumoperitoneum, and port placement is more difficult in such patients, with a conversion rate of up to 50% [5,6]. Additionally, they argue that the laparoscopic approach is less diagnostically reliable with sensitivity of only 18% and negative predictive value of 83%, where the diagnostic accuracy is dependent on the surgeon laparoscopy skills [6].

In the present case, the patient had already been submitted to a splenectomy in an emergent setting due to blunt abdominal trauma through a mid-line laparotomy. Given the likely presence of adhesions that could difficult the establishment of the pneumoperitoneum along with the risk of iatrogenic bowel lesions, the surgical team decided to perform a median laparotomy. This would reduce time-consuming adherence freeing and avoid eventual hollow viscus lesions related do pneumoperitoneum and port placement.

Five lacerations were detected, which were corrected according to the American Association for the Surgery of Trauma (AAST) guidelines. The outcome was very good, with a post-operative period without complications. Early diagnosis and surgical treatment are of extreme importance to obtain good results. A close surveillance with frequent abdominal evaluation together with hemodynamic status, are essential to detect early changes that indicate the need for surgical intervention.

This case report was written according to the Surgical CAse REport guidelines [11].

## 4 Conclusion

In conclusion, the diagnosis of small bowel injury is difficult, therefore a low threshold of suspicion is crucial to reduce morbidity and mortality [3,7]. The presence of hemodynamic instability, and/or abdominal tenderness and guarding after blunt abdominal trauma is classically accepted indications for emergent laparotomy. In this setting, even with negative imaging findings, serial clinical assessment is the main tool to decide whether the patient should undergo emergent exploratory laparotomy.

## Conflicts of interest statement

None.

## Funding

None.

## Ethical approval

The present study is exempt from ethical approval in our institution.

## Consent

Written consent from the patient was obtained.

## Author contribution

João Mendes Louro — Surgeon of the patient, conception of study design, review of clinical data, analysis and interpretation of data, drafting of the article, revising it critically for important intellectual content, final approval of the version to be submitted.

Carlos Costa Almeida — Surgeon of the patient, acquisition and analysis of data, revising it critically for important intellectual content, final approval of the version to be published.

Miguel Albano, Teresa Caroço, Luís Reis — Analysis and interpretation of data, revising it critically for important intellectual content.

## Registration of research studies

None.

## Guarantor

Carlos Eduardo Costa Almeida.

## Provenance and peer review

Not commissioned, externally peer reviewed.
